# The capacity and influencing factors of primary health care services in China

**DOI:** 10.1371/journal.pone.0331645

**Published:** 2025-09-02

**Authors:** Minghua Zhou

**Affiliations:** Department of administration office, Luzhou People’s Hospital, Luzhou, Sichuan, China; Northeastern University (Shenyang China), CHINA

## Abstract

**Objective:**

To analyze the capacity and influencing factors of primary health care services and to provide a scientific basis for promoting the development of primary health care services in China.

**Methods:**

The entropy weight technique for order preference by similarity to ideal solution (TOPSIS) and rank‒sum ratio (RSR) methods, which are based on the health resource density index (HRDI), were used to analyze the capacity of primary health care services, and multiple stepwise regression analysis was used to analyze the influencing factors.

**Results:**

Taking the HRDI of primary health care service capacity in 2021 as a reference, the six evaluation indicators of 14 regions, including Hebei, Liaoning, and Shanghai, were higher than the Chinese average. According to the entropy weight TOPSIS method, the average C-values of primary health care service capacity in China from 2017–2021 were 0.303, 0.313, 0.324, 0.331, and 0.326, respectively, with the C-values of regions such as Shanghai, Beijing, and Henan ranking in the top ten, whereas those of regions such as Xinjiang, Qinghai, and Tibet ranked in the bottom five. According to grade divisions by the RSR method, Tianjin, Shandong, Jiangsu, Shanghai, and Beijing were ranked at the good grade level; Xinjiang, Qinghai, Inner Mongolia, and Tibet at the poor grade level; and the remaining 22 regions at the medium grade level. According to the multivariate stepwise regression, population density, health technicians, and the number of beds per 1,000 people were the main factors affecting the capacity of primary health care services in China.

**Conclusion:**

The overall capacity of primary health care services in China is not high, and regional disparities are substantial. The capacity of primary health care services is better in Tianjin, Shandong, Jiangsu, Shanghai and Beijing and worse in Xinjiang, Qinghai, Inner Mongolia and Tibet. The population density and number of health technicians per 1,000 people are the main factors affecting the capacity of primary health care services in China.

## Introduction

The goal of promoting health care development is to provide equitable and accessible health care services, but there are substantial differences in health care services across regions in China. For many years, primary health care services have played an irreplaceable role as “health gatekeepers,” building a solid network of health care services and promoting the construction of hierarchical diagnoses and treatments [1]. Primary health care service capacity refers to the ability of primary health care institutions to provide basic health care services in communities, townships, and other grassroots areas, including prevention, medical treatment, rehabilitation, health promotion, and other services. These services aim to provide residents with convenient, accessible, and continuous basic health care. China continues to improve its primary health care service network and increase its capacity. Primary health care institutions account for nearly one-third of the health care workforce and provide more than half of the medical services and the vast majority of basic public health services. This system provides a fundamental safeguard for the health of urban and rural residents. Although the overall capacity of primary health care services in China has improved, there remain substantial disparities in the distribution of health care resources between urban and rural areas. The number of village health clinics and the proportion of outpatient visits at the primary care level have decreased. Additionally, there is a shortage of health care personnel in rural areas and inadequate implementation of the tiered health care system. China’s primary health care services will continue to focus on the primary level, with its core being the deployment of medical personnel; the comprehensive promotion of close-knit medical consortia; the strengthening of coordinated and collaborative efforts among county, township, and village medical services; and the continuous improvement of disease prevention and treatment capabilities of primary health care institutions. In October 2016, the State Council of the Central Committee of the Communist Party of China issued an outline of the “Healthy China 2030” plan, which emphasized focusing on rural and grassroots areas; promoting the equalization of basic public services in the field of health; gradually narrowing the gaps in basic health services and health levels between urban and rural areas and among various regions and different population groups; and achieving universal health coverage. In February 2023, the General Office of the Central Committee of the Communist Party of China and the General Office of the State Council issued the Opinions on Further Deepening Reforms to promote the Healthy Development of the Rural Medical and Health Care System, emphasizing the focus on the grassroots level and the improvement of the rural medical and health care system to expand access for rural people to more equitable and accessible, systematic and continuous health care services close to their home. Promoting the development of primary health care services and giving full play to the role of primary health care services as “health gatekeepers” is conducive to the better realization of equitable and accessible health care services for the people [2] and is crucial to narrowing the regional disparities in primary health care services and realizing health care for all.

Countries worldwide are taking proactive measures to achieve universal health coverage and strengthen primary health care service capabilities. At present, international studies on primary health care service capacity include Norway’s promotion of primary health care service development with patient participation [3], a survey of primary health care implementation in practice in Ghana [4], the current state of primary health care in Pakistan [5], and the state of primary health care centers in Saudi Arabia [6]. These studies have revealed insufficient allocation of primary health care resources and uneven regional distribution in many regions. Insufficient government investment, inadequate health care provider training, and limited medical equipment constrain the effectiveness of primary health care services, especially in rural areas. Specifically, studies on primary health care services in China can be divided into three main areas. First, studies on primary health care service capacity in China have focused on the rate of willingness to make a first visit to primary health care services in China [7], improving primary health care quality in China through training needs analysis [8], and the uneven primary health care supply of rural doctors and medical equipment in remote areas of China [9]. Second, studies on optimization measures for primary health care services in China, including supply‒demand balance and spatial distribution optimization of primary care facilities in the highland city of Lhasa [10], spatial accessibility evaluation and location optimization of primary health care in Shenzhen [11], and increasing physician volume in primary health care facilities, can help reduce hospital service utilization in China [12]. Third, studies on the impact of health care reform on primary health care services in China, including the role of the construction of a health care consortium on the allocation of human resources for primary care resources and its equity in China [13], the effects of vertical integration reform on primary health care institutions in China [14], the increased costs for primary care inpatients associated with vertical integration [15], and the perceptions of primary health care professionals regarding the vertical integration of the health care system in China [16], are needed. At present, relevant studies have focused primarily on surveys of primary health care service capacity, spatial optimization, and the impact of health care reform on primary health care services. However, the comprehensive evaluation of primary health care service capacity and its influencing factors still has room for improvement. China has a large population and a vast geographical area, with differing levels of economic development and uneven distributions of resources in different regions. The capacity of primary health care services in China and the influencing factors of primary health care services are worthy of in-depth study. On the basis of the principles of “maintaining the foundation, strengthening the grassroots level, and building a mechanism” for promoting medical system reform, it is necessary to prioritize the protection of people’s health, continually improve disease prevention and treatment, and enhance health management capabilities at the grassroots level to promote the sustainable development of primary health care services [17]. Analyzing primary health care service capacity in China and exploring its influencing factors are beneficial for guiding the allocation of health care resources in areas with weak primary health care resources. Formulating policies to promote the development of primary health care service capacity on the basis of actual conditions such as the population, economy, and society and promoting the deployment of medical service personnel to improve primary health care service capacity are important for reducing regional disparities, improving health equity, and achieving universal health coverage.

To promote the improvement of primary health care service capacity in China, this study used the entropy weight technique for order preference by similarity to ideal solution (TOPSIS) and rank–sum ratio (RSR) methods based on the health resource density index (HRDI) to analyze the capacity of primary health care services in China from 2017–2021 and used multiple stepwise regression analysis to analyze the influencing factors of primary health care service capacity in China from 2017–2021, thereby providing a scientific basis for narrowing the regional disparities in primary health care services and promoting the development of primary health care services in China.

## Methods

### Data sources

Data on the capacity and influencing factors of primary health care services in China were obtained from the China Health Statistics Yearbook from 2018–2022 and the China Statistical Yearbook from 2018–2022. Data may vary depending on the publication year, with the most recent data serving as the standard. A five-year time span was selected as the study period because it can adequately reflect changes in the development of primary health care service capacity. The scope of the study encompasses the data of 31 provincial-level administrative regions in mainland China (excluding Hong Kong, Macau, and Taiwan) from 2017 to 2021.

### Indicators

Primary health care institutions include community health service centers (stations), street health centers, township health centers, village health offices, outpatient clinics, and clinics (dispensaries). The number of institutions and beds are important indicators for measuring health care service capacity and the allocation of health care resources. These institutions provide the infrastructure necessary for health care services. Health technicians, licensed (assistant) physicians, and registered nurses are essential for achieving health care service goals and are the primary providers of professional medical services. General practitioners typically provide basic medical services in primary health care institutions and serve as “health gatekeepers” for residents. However, since the current evaluation of medical equipment uses mainly the number of devices costing more than 10,000 RMB, which does not reflect the actual situation of primary health care services, it has not been included in the evaluation indicators. On the basis of the principles of scientific rigor and data availability, the number of institutions, the number of beds, health technicians, licensed (assistant) physicians, registered nurses, and general practitioners in primary health care institutions were selected as evaluation indicators of service capacity. Health care services are influenced by factors such as the scale of medical care, population and economic conditions, and income and consumption levels. In terms of the scale of medical care, the numbers of beds, health technicians, and registered nurses represent the hardware and software resources of medical and health institutions. In terms of population and economic conditions, health resources are usually planned and allocated according to population size. Urbanization promotes the concentration of health resources, and per capita GDP is the main indicator of regional economic development. In terms of income and consumption levels, the disposable income of rural residents and their health care expenditures can be used to measure their overall standard of living. To analyze the factors affecting the capacity of primary health care services, the number of beds per 1,000 people, the number of health technicians per 1,000 people, and the ratio of doctors to nurses were selected as indicators for evaluating internal influences, and the population density, urbanization rate, per capita GDP, disposable income of rural residents, and health expenditure of rural residents were selected as indicators for evaluating external influences.

### Research methods

To comprehensively consider the influence of population and geographic factors on primary health care services, the entropy weight TOPSIS and RSR methods based on the HRDI were used to analyze the capacity of primary health care services in China from 2017–2021, and multiple stepwise regression analysis was used to analyze the influencing factors of primary health care service capacity in China from 2017–2021.

### Health resource density index (HRDI)

Primary health care services should consider not only the distribution of service recipients but also the convenience of service recipients in accessing health care. To analyze the combined impact of population and geographic factors on primary health care services, the HRDI was used to conduct a comprehensive analysis. The HRDI is the geometric mean of the product of health resources per 1,000 people and health resources per square kilometer, with larger values indicating a better distribution of health resources [18]. The formula is as follows:


$$\text{HRDI}=\sqrt{\frac{\text{Health}\;\text{resources}}{\text{Per}\;\text{1},\text{000}\;\text{people}}\times \frac{\text{Health}\;\text{resources}}{\text{Per}\;\text{square}\;\text{kilometer}}}$$

### Entropy weight method

The entropy weight method is an objective weighting method that avoids the bias caused by subjective weighting. It is commonly used in multi-indicator decision analysis [19]. The basic idea of the entropy weight method is to determine the weight of each evaluation index by calculating the amount of decision-making information that the index provides. This method can eliminate as much subjectivity in the weights of various factors as possible and more objectively reflects the amount of information contained in the data.

First, data standardization processing is performed. Construct the raw data matrix with n rows and m columns, where X_ij_ represents the data of the jth evaluation index of the ith region (i = 1,2,3,......,n; j = 1,2,3,...,m), and use the extreme value method to process the raw data with dimensionlessness and homotrendization. The formula is:


$${\text{r}}_{\text{ij}}=\frac{{\text{x}}_{\text{ij}}-{\text{x}}_{\text{min}(\text{j})}}{{\text{x}}_{\text{max}(\text{j})}-{\text{x}}_{\text{min}(\text{j})}}$$

$${\text{r}}_{\text{ij}}=\frac{{\text{x}}_{\text{max}(\text{j})}-{\text{x}}_{\text{ij}}}{{\text{x}}_{\text{max}(\text{j})}-{\text{x}}_{\text{min}(\text{j})}}$$

Next, calculate the entropy value E_j_ of each indicator, the coefficient of variation d_j_, and determine the indicator weights W_j_. The formula is as follows:


$${\text{E}}_{\text{j}}=-\text{k}\sum_{\text{i}=\text{1}}^{\text{n}}{\text{f}}_{\text{ij}\text{ln}{\text{f}}_{\text{ij}}},\;\text{\textbackslash{} where\textbackslash{} }{\text{f}}_{\text{ij}=\frac{{\text{r}}_{\text{ij}}}{\sum_{\text{i}=\text{1}}^{\text{n}}{\text{r}}_{\text{ij}}}}\text{\textbackslash{} k}=\frac{\text{1}}{\text{ln}\;\;\text{n}}$$

$${\text{d}}_{\text{j}\;=\;\text{1}-{\text{E}}_{\text{j}}}$$

$${\text{W}}_{\text{j}}=\frac{{\text{d}}_{\text{j}}}{\sum_{\text{j}=\text{1}}^{\text{m}}{\text{d}}_{\text{j}}},\;\text{where\textbackslash{} 0}\leq {\text{W}}_{\text{j}}\leq \text{1},\;\text{and\textbackslash{} }\sum_{\text{j}=\text{1}}^{\text{m}}{\text{d}}_{\text{j}}=\text{1}$$

### TOPSIS method

The TOPSIS method accurately reflects the relative order of advantages and disadvantages between the evaluation indicators by calculating the relative distance between each evaluation indicator and the ideal optimal solution [20]. The TOPSIS method effectively retains original information and responds sensitively to differences in the original indicators.

First, the original data normalization processing is performed. Construct the decision matrix and normalization process for the original data, and obtain the normalized matrix Z_ij_. The formula is as follows:


$${\text{Z}}_{\text{ij}}=\frac{{\text{X}}_{\text{ij}}}{\sqrt{\sum_{\text{i}=\text{1}}^{\text{n}}{\text{X}}_{\text{ij}}^{\text{2}}}}$$

Second, calculate the weighted distance of each evaluation index. According to the normalization matrix and the weight of each evaluation index, construct the weighted decision matrix Y to find the optimal vector y_j_^+^ and the worst vector y_j_^-^. Then calculate the distance between each index and the optimal vector and the worst vectors D_i_^+^ and D_i_^-^, respectively. The formula is as follows:


$$Y={\left(y_{ij}\right)}_{n\times m}={{(Z}_{ij}W_{j})}_{n\times m}$$

$$y_{i}^{+}=\text{max}\left\{y_{1j},\;y_{2j},\ldots ,y_{nj}\right\},\;y_{i}^{-}=\text{min}\left\{y_{1j},\;y_{2j},\ldots ,y_{nj}\right\}$$

$${\text{D}}_{\text{i}}^{+}=\sqrt{{\sum_{\text{j}=\text{0}}^{\text{m}}\left[{\text{w}}_{\text{j}}\left({\text{y}}_{\text{ij}}-{\text{y}}_{\text{i}}^{+}\right)\right]}^{\text{2}}\text{\textbackslash{} \textbackslash{} \textbackslash{} \textbackslash{} }}{,\;\text{D}}_{\text{i}}^{-}=\sqrt{{\sum_{\text{j}=\text{0}}^{\text{m}}\left[{\text{w}}_{\text{j}}\left({\text{y}}_{\text{ij}}-{\text{y}}_{\text{i}}^{-}\right)\right]}^{\text{2}}}$$

Third, calculate the relative proximity C-value: the greater the C-value is, the better the capacity of primary health care services in the region. The formula is as follows:


$${\text{C}}_{\text{i}}=\frac{{\text{D}}_{\text{i}}^{+}}{{\text{D}}_{\text{i}}^{+}+{\text{D}}_{\text{i}}^{-}},\;\text{0}\leq {\text{C}}_{\text{i}}\leq \text{1}$$

### RSR method

The RSR method is a nonparametric evaluation method that uses rank order to replace original data, effectively compensating for the shortcomings of the TOPSIS method in terms of classification and enabling the classification of evaluation results on the basis of their quality. The basic idea of the RSR method is to obtain dimensionless rank‒sum ratios through rank transformation of multiple indicators; the RSR values are then used to rank the advantages and disadvantages of the evaluation objects in a graded order [21].

First, the RSR distribution values from 2017 to 2021 calculated by the entropy weight RSR method were sorted from smallest to largest, cumulative frequency P values were calculated, and P values were converted to probit values.

Second, the RSR distribution values were used as the dependent variable, and the probit values were used as the independent variables to construct a linear regression equation for testing and to calculate the RSR estimates.

Finally, the evaluation subjects were graded.

### Multiple stepwise regression analysis

Stepwise regression analysis is a statistical method based on linear regression. The basic idea is to introduce variables one by one and, after introducing a new variable, test each of the old variables in the regression model individually. Variables that are considered meaningless are deleted until no new variables are introduced and no old variables are deleted. This process ensures that every variable in the regression model is meaningful. Multiple stepwise regression analysis is a statistical method for establishing a linear or nonlinear mathematical model of quantitative relationships among multiple variables by considering one variable as the dependent variable and one or more other variables as independent variables [22]. Multiple stepwise regression analysis was performed with the C value as the dependent variable and the eight influencing factor evaluation indicators as the independent variables, with a test level of α = 0.05. The mathematical model of the regression equation is as follows:


$$\text{Y}=\beta_{\text{0}}+\beta_{\text{1}}{\text{x}}_{\text{1}}+\beta_{\text{2}}{\text{x}}_{\text{2}}+\ldots +\beta_{\text{k}}{\text{x}}_{\text{k}}+\epsilon$$

β_0_ is a constant term, β_i_ (i = 1,2,......,k) denotes the average change in the value of the corresponding Y for a change of one unit in X_i_ when the independent variables other than X_i_ are fixed, and ε is a random error term.

### Statistical analyses

Excel 2010 was used for data collection, and SPSS 22.0 was used for data analysis.

## Results

### HRDI for primary health care services in China, 2017–2021

**Fig 1:**
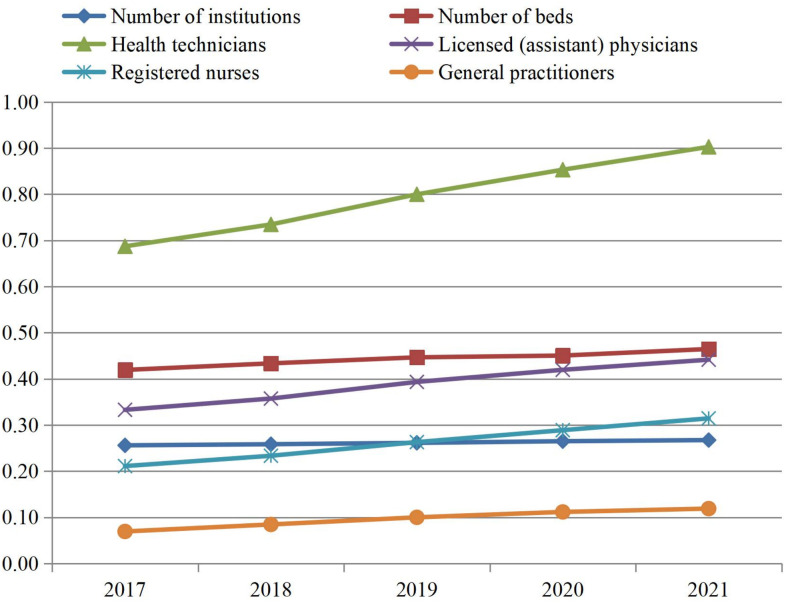
HRDI for primary health care services in China, 2017–2021.

The HRDI for the number of institutions, number of beds, health technicians, licensed (assistant) physicians, registered nurses, and general practitioners increased from 0.256, 0.419, 0.687, 0.333, 0.211, and 0.069 in 2017 to 0.267, 0.465, 0.903, 0.442, 0.314, and 0.119 in 2021, respectively. Owing to administrative division adjustments, primary health care institutions underwent restructuring or mergers, which resulted in slow growth in the number of institutions. As health care reforms have emphasized a focus on primary care, health care human resources have grown rapidly, with the number of general practitioners and registered nurses increasing rapidly (Fig 1).

### HRDI for primary health care services in China in 2021

In 2021, the HRDI values of the number of institutions, the number of beds, health technicians, licensed (assistant) physicians, registered nurses and general practitioners of primary health care services in China were 0.267, 0.465, 0.903, 0.442, 0.314 and 0.119, respectively. Taking the HRDI of primary health care service capacity in China in 2021 as a reference, the six evaluation indicators in 14 regions, including Hebei, Liaoning, and Shanghai, were higher than the Chinese average, and the six evaluation indicators in 7 regions, including Inner Mongolia, Heilongjiang, and Tibet, were lower than the Chinese average (Table 1). In terms of the HRDI for the number of institutions, regions such as Hebei and Shandong had relatively high scores, whereas regions such as Xinjiang and Qinghai had relatively low scores. In terms of the HRDI of the number of beds, regions such as Henan and Chongqing had relatively large numbers of beds, whereas regions such as Qinghai and Tibet had relatively small numbers of beds. The numbers of institutions and beds were concentrated in densely populated areas, with sparsely populated areas with large geographical areas having relatively few institutions and beds. The HRDI of health technicians, licensed (assistant) physicians, and registered nurses was relatively high in regions such as Shanghai and Beijing, whereas it was relatively low in regions such as Xinjiang, Tibet, and Qinghai. This difference may be due to the influence of socioeconomic development. Regions such as Shanghai have a high level of socioeconomic development, and the public in these regions has a high demand for health care services, leading to greater health care resource allocation.

**Table 1:** HRDI for primary health care services in China in 2021.

Regions	Number of institutions	Number of beds	Health technicians	Licensed (assistant) physicians	Registered nurses	General practitioners
Beijing	0.516	0.277	3.860	1.854	1.387	0.491
Tianjin	0.429	0.459	2.417	1.310	0.680	0.439
Hebei	0.718	0.673	1.346	0.862	0.335	0.206
Shanxi	0.529	0.510	1.014	0.577	0.321	0.101
Inner Mongolia	0.143	0.165	0.361	0.188	0.110	0.037
Liaoning	0.391	0.498	0.998	0.518	0.364	0.151
Jilin	0.359	0.305	0.958	0.497	0.334	0.123
Heilongjiang	0.158	0.275	0.483	0.255	0.142	0.058
Shanghai	0.395	1.085	4.409	2.012	1.780	0.745
Jiangsu	0.350	1.099	2.454	1.204	0.882	0.519
Zhejiang	0.398	0.359	2.129	1.097	0.703	0.282
Anhui	0.299	0.886	1.492	0.769	0.552	0.185
Fujian	0.381	0.520	1.326	0.634	0.458	0.162
Jiangxi	0.406	0.754	0.965	0.448	0.329	0.111
Shandong	0.649	0.963	2.028	1.042	0.678	0.284
Henan	0.588	1.180	1.641	0.893	0.498	0.264
Hubei	0.334	0.973	1.325	0.604	0.518	0.121
Hunan	0.450	1.055	1.296	0.645	0.471	0.152
Guangdong	0.365	0.502	1.788	0.812	0.706	0.258
Guangxi	0.298	0.746	1.192	0.466	0.443	0.120
Hainan	0.310	0.536	1.406	0.615	0.596	0.150
Chongqing	0.394	1.122	1.615	0.771	0.598	0.174
Sichuan	0.382	0.738	1.046	0.495	0.372	0.103
Guizhou	0.333	0.656	1.056	0.430	0.372	0.113
Yunnan	0.186	0.464	0.832	0.326	0.351	0.069
Tibet	0.100	0.064	0.118	0.055	0.027	0.007
Shaanxi	0.386	0.471	1.141	0.502	0.339	0.154
Gansu	0.243	0.330	0.550	0.244	0.196	0.074
Qinghai	0.092	0.087	0.186	0.085	0.056	0.026
Ningxia	0.219	0.200	0.829	0.367	0.313	0.084
Xinjiang	0.075	0.192	0.216	0.092	0.073	0.024
China	0.267	0.465	0.903	0.442	0.314	0.119

### Indicator weights of primary health care service capacity in China

When the HRDI was used to calculate the weights of the evaluation indicators for 2017–2021, the weights of the number of institutions, the number of beds, health technicians, licensed (assistant) physicians, registered nurses, and general practitioners in 2017 were 11.779%, 12.434%, 14.758%, 15.785%, 16.032%, and 29.212%, respectively, and the weights of the number of institutions, the number of beds, health technicians, licensed (assistant) physicians, registered nurses, and general practitioners in 2021 were 11.629%, 14.128%, 17.001%, 17.424%, 17.597%, and 22.221%, respectively (Table 2).

**Table 2:** Indicator weights of primary health care service capacity in China.

Year	Number of institutions	Number of beds	Health technicians	Licensed (assistant) physicians	Registered nurses	General practitioners
2017	11.779	12.434	14.758	15.785	16.032	29.212
2018	11.729	12.527	15.417	16.284	16.877	27.166
2019	10.955	12.518	16.099	16.846	17.377	26.205
2020	11.995	13.830	16.389	16.828	17.194	23.764
2021	11.629	14.128	17.001	17.424	17.597	22.221

### C value and ranking of the comprehensive evaluation of primary health care service capacity in China by the entropy weight TOPSIS method

The entropy weight TOPSIS method based on the HRDI was used to calculate the C value of the primary health care service capacity in China from 2017–2021. The C values of Shanghai, Beijing, Jiangsu, Shandong, Tianjin, Zhejiang, and Henan were greater and ranked in the top ten; the C values of Xinjiang, Qinghai, Inner Mongolia, and Tibet were smaller and ranked in the bottom five. From 2017–2021, the average C values of primary health care service capacity in China were 0.303, 0.313, 0.324, 0.331 and 0.326, respectively (Table 3). The primary health care service capacity in China has gradually improved overall, which is consistent with the trend of improved health resource allocation in hospitals and primary health care centers. This finding indicates that the health care reforms implemented since 2009 have been somewhat successful [23].

**Table 3:** C values and rankings of the comprehensive evaluation of primary health care service capacity in China by entropy weight TOPSIS method.

Regions	2017		2018		2019		2020		2021	
	C value	Ranking	C value	Ranking	C value	Ranking	C value	Ranking	C value	Ranking
Beijing	0.673	2	0.687	2	0.689	2	0.685	2	0.660	2
Tianjin	0.479	5	0.500	4	0.499	5	0.501	5	0.512	5
Hebei	0.390	8	0.404	9	0.416	10	0.422	10	0.424	8
Shanxi	0.326	13	0.317	13	0.316	14	0.316	15	0.308	16
Inner Mongolia	0.066	28	0.067	28	0.066	29	0.068	28	0.067	28
Liaoning	0.281	18	0.294	17	0.284	18	0.287	20	0.278	21
Jilin	0.196	23	0.208	23	0.210	23	0.239	23	0.231	23
Heilongjiang	0.105	27	0.106	27	0.115	28	0.110	27	0.108	27
Shanghai	0.824	1	0.824	1	0.838	1	0.836	1	0.844	1
Jiangsu	0.529	3	0.634	3	0.632	3	0.634	3	0.605	3
Zhejiang	0.498	4	0.466	6	0.464	6	0.455	7	0.421	9
Anhui	0.294	14	0.309	14	0.325	13	0.392	11	0.381	12
Fujian	0.287	17	0.298	15	0.301	16	0.314	16	0.309	14
Jiangxi	0.292	15	0.288	18	0.293	17	0.309	17	0.306	17
Shandong	0.463	6	0.489	5	0.503	4	0.518	4	0.526	4
Henan	0.416	7	0.434	7	0.437	7	0.469	6	0.495	6
Hubei	0.355	11	0.366	12	0.365	12	0.377	13	0.366	13
Hunan	0.376	9	0.375	11	0.420	9	0.427	9	0.403	10
Guangdong	0.353	12	0.376	10	0.380	11	0.391	12	0.386	11
Guangxi	0.263	19	0.269	19	0.283	19	0.307	19	0.302	19
Hainan	0.237	22	0.240	22	0.247	22	0.307	18	0.308	15
Chongqing	0.371	10	0.404	8	0.427	8	0.443	8	0.436	7
Sichuan	0.291	16	0.298	16	0.310	15	0.326	14	0.305	18
Guizhou	0.246	20	0.253	20	0.262	20	0.286	21	0.278	20
Yunnan	0.158	24	0.168	24	0.182	24	0.197	24	0.189	24
Tibet	0.010	31	0.010	31	0.010	31	0.011	31	0.013	31
Shaanxi	0.243	21	0.246	21	0.253	21	0.258	22	0.275	22
Gansu	0.152	25	0.149	25	0.149	27	0.153	26	0.148	26
Qinghai	0.024	30	0.024	30	0.169	25	0.021	30	0.021	30
Ningxia	0.132	26	0.149	26	0.152	26	0.163	25	0.154	25
Xinjiang	0.047	29	0.047	29	0.050	30	0.043	29	0.047	29
Average	0.303	—	0.313	—	0.324	—	0.331	—	0.326	—

### Frequency distributions and probability values of the RSR method for primary health care service capacity in China

The entropy weight method was used to weight the C values, which were 20.758%, 20.887%, 18.081%, 19.927%, and 20.346% for 2017–2021, respectively. The RSR distribution values calculated by the entropy weight RSR method from 2017–2021 were ranked from smallest to largest; the number of times, cumulative number of times, evaluation rank, and cumulative frequency P values were compiled; and the last item of the P value was corrected by (1–1/4n) × 100%. The P values were converted to probit values by referring to the table of comparisons of percentage and probability units. Linear regression analysis was performed using the RSR distribution values as the dependent variable and the probit values as the independent variables to calculate the estimated RSR values, and the regression equation was as follows: y = −0.937 + 0.286Probit, where R^2^ = 0.964, F = 768.082, and P < 0.001; this regression equation was statistically significant (Table 4).

**Table 4:** Frequency distribution and probability value of RSR method of primary health care service capacity in China.

Regions	RSR distribution values	Number of times	Cumulative number of times	Evaluation rank	Cumulative frequency	Probit	RSR estimated values
Tibet	0.032	1	1	1	3.23	3.151	−0.035 1
Xinjiang	0.091	1	2	2	6.45	3.482	0.059 6
Qinghai	0.094	1	3	3	9.68	3.700	0.121 9
Inner Mongolia	0.123	1	4	4	12.90	3.869	0.170 3
Heilongjiang	0.155	1	5	5	16.13	4.011	0.210 9
Gansu	0.201	1	6	6	19.35	4.135	0.246 4
Ningxia	0.207	1	7	7	22.58	4.247	0.278 5
Yunnan	0.258	1	8	8	25.81	4.351	0.308 1
Jilin	0.290	1	9	9	29.03	4.448	0.335 8
Shaanxi	0.342	1	10	10	32.26	4.540	0.362 1
Guizhou	0.381	1	11	11	35.48	4.628	0.387 4
Hainan	0.394	1	12	12	38.71	4.713	0.411 8
Guangxi	0.419	1	13	13	41.94	4.796	0.435 7
Liaoning	0.426	1	14	14	45.16	4.878	0.459 1
Jiangxi	0.491	1	15	15	48.39	4.960	0.482 3
Sichuan	0.522	1	16	16	51.61	5.040	0.505 5
Fujian	0.529	1	17	17	54.84	5.122	0.528 7
Shanxi	0.575	1	18	18	58.06	5.204	0.552 2
Anhui	0.619	1	19	19	61.29	5.287	0.576 0
Hubei	0.639	1	20	20	64.52	5.372	0.600 5
Guangdong	0.671	1	21	21	67.74	5.460	0.625 7
Hunan	0.722	1	22	22	70.97	5.552	0.652 0
Hebei	0.743	1	23	23	74.19	5.649	0.679 7
Chongqing	0.767	1	24	24	77.42	5.753	0.709 3
Henan	0.819	1	25	25	80.65	5.865	0.741 4
Zhejiang	0.826	1	26	26	83.87	5.989	0.777 0
Tianjin	0.878	1	27	27	87.10	6.131	0.817 6
Shandong	0.883	1	28	28	90.32	6.300	0.866 0
Jiangsu	0.935	1	29	29	93.55	6.518	0.928 3
Beijing	0.968	1	30	30	96.77	6.849	1.022 9
Shanghai	1.000	1	31	31	99.19	7.406	1.182 4

### Grade results of the RSR method for primary health care service capacity in China

**Fig 2:**
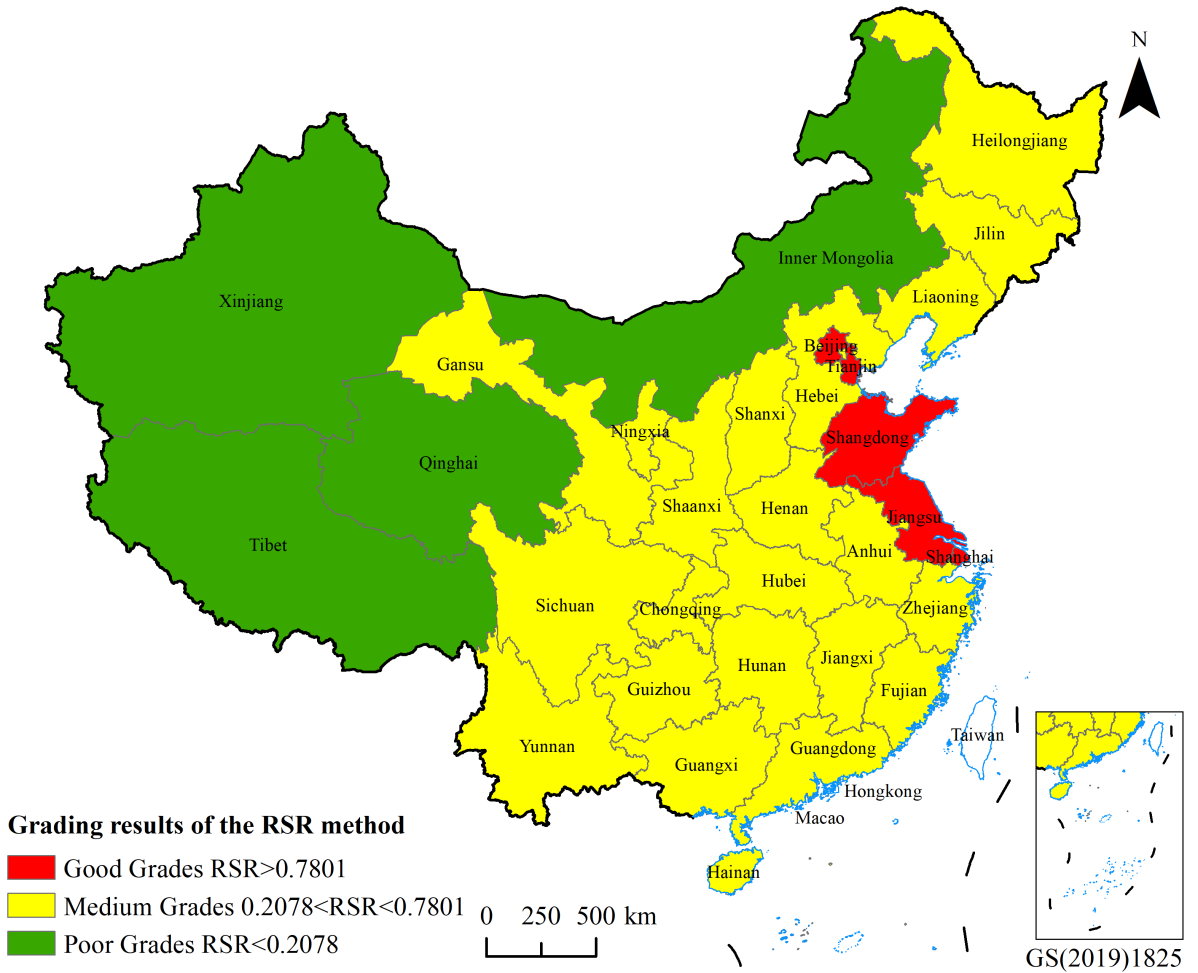
Grade results of the RSR method for primary health care service capacity in China. The base map is derived from the National Natural Resources and Geospatial Basic Information Database of the PRC (https://www.sgic.net.cn/portal/index.html#/Home), review number GS(2019)1825, and these shapes are licensed without need for permission.

According to the RSR optimal grading method and grading table, the primary health care service capacity in China can be divided into three grade levels, with 5 regions in the good grade, 22 regions in the medium grade, and 4 regions in the poor grade. Tianjin, Shandong, Jiangsu, Shanghai and Beijing were at the good grade level; Xinjiang, Qinghai, Inner Mongolia and Tibet were at the poor grade level; and the remaining regions were at the medium grade level (Fig. 2). Although primary health care resources in China are expanding, the issue of uneven distribution has yet to be addressed. Key priorities for the development of primary health care include strengthening the primary health care workforce and expanding access to primary health care services [24].

### Stepwise regression results of the factors influencing the C value of the primary health care service capacity in China

Multiple stepwise regression analysis was conducted with the C value as the dependent variable and the 8 evaluation indicators of influencing factors as the independent variables. According to the results of the F test, P < 0.001 indicates that the overall regression coefficient is not zero; that is, there is a regression relationship between the variables. On the basis of the performance of variable multicollinearity, all the VIF values are less than 5, indicating that the model is well constructed and does not have multicollinearity issues. Population density was the main factor affecting primary health care service capacity in China from 2017 to 2021. In addition to 2019, the number of health technicians per 1,000 people was also a main factor affecting primary health care service capacity in China. Since 2020, the number of beds per 1,000 people has become one of the main factors affecting primary health care service capacity in China (Table 5).

**Table 5:** Stepwise regression results of the influencing factors of the C value of the primary health care service capacity in China.

Year	Influencing factors	Nonstandardized coefficient		Standardized coefficient	*t*	*p*	VIF	*R* ^2^	Adjusted*R*^2^	F
		B	Standard errors	*Beta*						
2017	Constant	−0.081	0.115	–	−0.704	0.487	–	0.778	0.762	F = 49.142, *P* < 0.001
	Health technicians per 1,000 people	0.154	0.066	0.218	2.340	0.027	1.092			
	Population density	0.000	0.000	0.794	8.538	<0.001	1.092			
2018	Constant	−0.107	0.122	–	−0.875	0.389	–	0.764	0.748	F = 45.431, *P* < 0.001
	Health technicians per 1,000 people	0.163	0.066	0.241	2.484	0.019	1.122			
	Population density	0.000	0.000	0.765	7.869	<0.001	1.122			
2019	Constant	0.205	0.023	–	9.011	<0.001	–	0.719	0.709	F = 74.033, *P* < 0.001
	Population density	0.000	0.000	0.848	8.604	<0.001	1.000			
2020	Constant	−0.250	0.139	–	−1.794	0.084	–	0.780	0.756	F = 32.001, *P* < 0.001
	Number of beds per 1,000 people	0.101	0.046	0.216	2.176	0.038	1.211			
	Health technicians per 1,000 people	0.157	0.057	0.260	2.752	0.010	1.095			
	Population density	0.000	0.000	0.842	8.226	<0.001	1.288			
2021	Constant	−0.243	0.140	–	−1.732	0.095	–	0.790	0.766	F = 33.772, *P* < 0.001
	Number of beds per 1,000 people	0.092	0.044	0.208	2.114	0.044	1.245			
	Health technicians per 1,000 people	0.146	0.054	0.257	2.717	0.011	1.149			
	Population density	0.000	0.000	0.841	8.225	<0.001	1.340			

## Discussion

Both the HRDI and the entropy weight TOPSIS methods reveal obvious regional differences in the capacity of primary health care services in China. We offer three reasons for these differences. First, the levels of population and economic development have a substantial impact on primary health care services. Areas with large populations have relatively more financial input, and areas with better economic development levels have relatively more financial security, thus leading to better primary health care service capacity [25]. Most of the areas with large C values are in the eastern regions; notably, Henan is among the top ten because it has always been a populous province with a large population that has a strong demand for health services, resulting in relatively good primary health care services. Second, health policies in different areas are uneven in their support for primary health care services. Areas that have strengthened the construction of rural health service systems and promoted the investment of medical resources have relatively better primary health care service capacity [26]. For example, areas such as Hubei and Sichuan have focused on strengthening the construction of primary health care service systems [27] and have achieved better results in terms of financial investment and the standardization of township health centers and village health offices, with a relatively strong capacity for primary health care services. Third, infrastructure development has affected the development of primary health care services [28]. Compared with that in the eastern regions, infrastructure development in the western regions is characterized by a large geographical radius, complex terrain, high medical costs, insufficient allocation of health resources to the primary areas, and a wide gap between medical conditions in the primary and urban areas, which has limited the development of primary health care services. Therefore, all regions should emphasize the construction of primary health care service systems, increase financial investments, improve health care infrastructure in rural areas, and supplement health care personnel in rural areas, thereby promoting the growth of primary health care services [29]. Urban areas should support rural areas by improving remote medical service networks and facilitating medical treatment through methods such as mobile and village-based services.

The average C value of primary health care service capacity in China did not exceed 0.4 and decreased in 2021, indicating that the overall level of primary health care service capacity in China is not high. Under the basic principle of “maintaining the foundation, strengthening the grassroots level, and building a mechanism”, policies such as graded diagnosis and treatment, the construction of medical consortia, and the signing up of family doctors have been introduced, thereby promoting the development of primary health care service capacity [30]. The average C value of primary health care service capacity in China gradually increased from 2017 to 2020, which is a manifestation of the gradual improvement in primary health care service capacity. Although the capacity of primary health care services has developed, the overall level is not high, and we believe that there are two reasons for this. On the one hand, there are still deficiencies in the allocation of health resources in primary areas. With policy support, the implementation of hardware, such as health centers and beds in primary areas, has improved, but major deficiencies in health technicians, medical facilities and equipment, and the supply of medicines have occurred, leading to a lack of capacity in primary health care services [31]. Influenced by factors such as better remuneration packages, opportunities for career development, and convenient transportation in large hospitals and urban areas, health workers in primary areas are easily lost to other areas, making it difficult to develop primary health care services [32]. On the other hand, the development of primary health care services has been affected by the gradual decline in the number of consultations and the outflow of medical insurance from primary areas. With the development of urbanization and the migration of the population to urban areas, coupled with the influence of the traditional concept of “going to the big hospitals”, primary areas are suffering from a crisis of patient confidence, leading to a decline in the number of consultations and an outflow of medical insurance, which has made it difficult to sustain the development of primary health care [33]. Therefore, it is necessary to continue to strengthen the allocation of health resources in rural areas, improve the departmental structure and equipment of rural health care institutions, make use of traditional Chinese medicine, and enhance rural health care services to keep patients in rural areas [34]. It is also necessary to strengthen health education and awareness, reduce service distances, and enable the general public to access safe and effective basic health care services nearby.

The capacity of primary health care services in Tianjin, Shandong, Jiangsu, Shanghai and Beijing is ranked at the good grade level, indicating superior capacity of primary health care services in these areas. We believe that there are two reasons for this. On the one hand, these areas are all in the eastern region, where the capacity of primary health care services is better developed. The eastern region has a large population, a better level of economic development, a greater allocation of health resources in the primary area, and a higher level of health awareness among the population, resulting in a better capacity for primary health care [35]. On the other hand, health reform and the development of information technology in these areas have promoted the development of primary health care service capacity. These areas have been at the forefront of health reform, with the earlier establishment of medical consortia and the signing up of family doctors and other services [36], health information technology improving the coverage of health services, and the gradual development of social medical institutions supplementing the health care market, thus helping improve the capacity of primary health care services. Therefore, areas with better primary health care service capacity should make full use of the spillover effect of resource allocation to achieve the coordinated development of primary health care resources and the health care market. It is also necessary to strengthen support for areas with weak primary health care services, promote family doctor contract services, and utilize technologies such as telemedicine and artificial intelligence-assisted diagnosis. These measures will provide residents with comprehensive, full-lifecycle health care services and improve the equity of primary health care services. Xinjiang, Qinghai, Inner Mongolia and Tibet are ranked at the poor grade level, indicating weak capacity of primary health care services in these areas. These areas are sparsely populated, with insufficient financial input, inadequate allocation of health resources, high health service costs, and limited health resources concentrated in urban areas [37], resulting in inadequate allocation of health resources in primary areas and poorer health service capacity in primary areas [38]. Therefore, these areas should further improve the construction of the rural health system through various measures, such as providing special programs for college students to become village doctors, conducting staff training and further education, and promoting the deployment of urban hospital personnel to rural areas to continuously expand the primary health care workforce. These areas should also strengthen the construction of primary health care institutions, increase the coverage of village health clinics, reduce the distance for the public to access health care services, and thereby improve the utilization efficiency of primary health care services [39].

Through stepwise regression analysis, population density was found to be the main factor affecting primary health care capacity in China [40]. Health care planning in China usually uses the number of health resources per 1,000 people as the planning target, which results in a relatively adequate allocation of health resources in densely populated areas but makes it difficult for sparsely populated and geographically large areas to obtain adequate health resources [41]. This problem is a concrete manifestation of the fact that areas with better primary health care capacity are concentrated in the eastern region, and those with weaker capacity are concentrated in the western region. Health technicians per 1,000 people are also among the main factors affecting primary health care service capacity in China, indicating that health technicians play an important role in the allocation of primary health care resources. Health technicians are the main providers of primary health care services and are thus the most creative and dynamic factor, with a direct impact on the quality and efficiency of primary health care services [42]. Health technicians take on the role of using medical resources such as beds and equipment, as well as providing health services and retaining patients, which has a considerable impact on the capacity of health services [43]. Since 2020, the number of beds per 1,000 people has become one of the main factors affecting primary health care service capacity in China, indicating that beds have gradually become a relatively scarce medical resource with the impact of the growing demand for medical services. Adequate beds are conducive to meeting the needs of patients, whereas improving the efficiency of bed utilization is conducive to improving the service level of hospitals. Therefore, it is necessary to continuously optimize the allocation of primary health care resources, especially beds, on the basis of factors such as population density, distance to services, and resident needs. It is also important to improve the technical capacity and service level of health technicians in primary areas [44] and ensure that primary health care services fully play the role of “health gatekeepers”.

## Limitations

Although we used the entropy weight TOPSIS and RSR methods based on the HRDI to analyze the capacity of primary health care services in China and multiple stepwise regression analysis to analyze the influencing factors from 2017 to 2021, this study has several limitations. First, although we considered the influence of population and geographic area factors on primary health care services, we did not analyze regional differences. Additionally, we did not distinguish between differences in service capacity and influencing factors in the eastern, central, and western regions. Second, although we tried to include as many evaluation indicators as possible and were limited by data availability, we did not include health input indicators. Third, we conducted a comprehensive analysis of the capacity of primary health care services and did not cover the aspect of people’s demand for primary health care services.

## Conclusions

To promote the development of primary health care services in China, we used the entropy weight TOPSIS and RSR methods based on the HRDI to analyze the capacity of primary health care services in China from 2017 to 2021 and multiple stepwise regression analysis to analyze the influencing factors from 2017 to 2021. The study revealed that there are significant regional differences in primary health care service capacity in China and that the overall level of primary health care service capacity in China is not high. Tianjin, Shandong, Jiangsu, Shanghai, and Beijing have relatively strong primary health care service capacity, whereas Xinjiang, Qinghai, Inner Mongolia, and Tibet have relatively weak primary health care service capacity. Population density, health technicians, and the number of beds per 1,000 people are the main factors affecting the capacity of primary health care services in China. We suggest that it is necessary to further improve the construction of the primary health care service system, improve the departmental structure and equipment configuration of primary health care institutions, and continuously supplement and optimize the allocation of primary health care resources on the basis of factors such as population density, service distance, and resident needs to shorten the distance for the general public to access health care services and improve the utilization efficiency of primary health care services. Various measures should be taken to strengthen the primary health care workforce, such as implementing a special program for college students to become rural doctors, conducting staff training and further education, and promoting the deployment of urban hospital personnel in rural areas. Technologies such as telemedicine and artificial intelligence-assisted diagnosis should be applied to improve the technical capacity and service level of health technicians in primary areas and to ensure that primary health care services fully play the role of “health gatekeepers”.

## Supporting information

S1 Table Data on the capacity and influencing factors of primary health care services in China, 2017–2021. (XLSX)

## References

[1] Zhu D, Shi X, Chen S, Ye X, Nicholas S, He P. The role of primary health care in improving health status, financial protection and health equity in the context of China’s health system reform. Int J Health Plann Manage. 2024;39(2):311–28. doi: 10.1002/hpm.3722 37915063

[2] Cai C, Xiong S, Millett C, Xu J, Tian M, Hone T. Health and health system impacts of China’s comprehensive primary healthcare reforms: a systematic review. Health Pol Plan. 2023,38(9):1064–78. doi: 10.1093/heapol/czad058 PMC10566320 37506039

[3] Sandvin Olsson AB, Stenberg U, Haaland-Øverby M, Slettebø T, Strøm A. Enabling primary healthcare service development with patient participation: a qualitative study of the internal facilitator role in Norway. Prim Health Care Res Dev. 2023;24. doi: 10.1017/s1463423623000488 PMC10539736 37753659

[4] Appiah-Agyekum NN. Primary healthcare implementation in practice: evidence from primary healthcare managers in Ghana. African J Prim Health Care Family Med. 2020;12(1). doi: 10.4102/phcfm.v12i1.2183 PMC7284167 32501025

[5] Ahmed SH, Zahid M, Waseem S, Zafar A, Shaikh TG, Sabri T, et al. The current state of primary healthcare in Pakistan: a way forward for low-to-middle income countries. Prim Health Care Res Dev. 2024;25. doi: 10.1017/s1463423624000549 PMC11569849 39478436

[6] Kattan W. The state of primary healthcare centers in Saudi Arabia: a regional analysis for 2022. PLoS One. 2024;19(9):e0301918. doi: 10.1371/journal.pone.0301918 39250494 PMC11383242

[7] Liu C, Qiu L, Wang H. Willingness rate of the first visit to primary healthcare services and the associated factors in China: a meta-analysis. Aust J Prim Health. 2022;28(6):459–68. doi: 10.1071/py21296 35858635

[8] Liu B, Xue Q, Li X, Sun J, Rao Z, Zou G, et al. Improving primary healthcare quality in China through training needs analysis. Sci Rep. 2024;14(1):30146. doi: 10.1038/s41598-024-81619-0 39627421 PMC11615283

[9] Shan L, Gan Y, Yan X, Wang S, Yin Y, Wu X. Uneven primary healthcare supply of rural doctors and medical equipment in remote China: community impact and the moderating effect of policy intervention. Int J Equity Health. 2024;23(1):97. doi: 10.1186/s12939-024-02183-7 38735959 PMC11089803

[10] Yu Y, Zhou R, Qian L, Yang X, Dong L, Zhang G. Supply-demand balance and spatial distribution optimization of primary care facilities in highland cities from a resilience perspective: a study of Lhasa, China. Front Public Health. 2023;11. doi: 10.3389/fpubh.2023.1131895 PMC10032525 36969676

[11] Chen L, Zeng H, Wu L, Tian Q, Zhang N, He R, et al. Spatial accessibility evaluation and location optimization of primary healthcare in china: a case study of shenzhen. Geohealth. 2023;7(5):e2022GH000753. doi: 10.1029/2022GH000753 37200630 PMC10187614

[12] Li X, Xu H, Du F, Zhu B, Xie P, Wang H, et al. Does increasing physician volume in primary healthcare facilities under the hierarchical medical system help reduce hospital service utilisation in China? A fixed-effects analysis using province-level panel data. BMJ Open. 2023;13(2):e066375. doi: 10.1136/bmjopen-2022-066375 36822814 PMC9950906

[13] Li S, Liao C, Zhang S. The role of construction of healthcare consortium on the allocation of human resources for primary care resources and its equity in China: a quantitative study. PLoS One. 2024;19(8):e0304934. doi: 10.1371/journal.pone.0304934 39213319 PMC11364225

[14] Yuan S, Fan F, Zhu D. Effects of vertical integration reform on primary healthcare institutions in china: evidence from a longitudinal study. Int J Health Policy Manag. 2022;11(9):1835–43. doi: 10.34172/ijhpm.2021.93 34634876 PMC9808208

[15] Tan H, Zhang X, Peng X, Guo D, Chen Y. Does vertical integration increase the costs for primary care inpatients? Evidence from a national pilot programme in China. Arch Public Health. 2024;82(1):136. doi: 10.1186/s13690-024-01378-2 39187907 PMC11346275

[16] Yuan S, Fan F, van de Klundert J, van Wijngaarden J. Primary healthcare professionals’ perspective on vertical integration of healthcare system in China: a qualitative study. BMJ Open. 2022;12(2):e057063. doi: 10.1136/bmjopen-2021-057063 35105599 PMC8808441

[17] Xu R, Xu C, Wu L, Xie X, Mu T. Spatial accessibility and equity of primary healthcare in Zhejiang, China. Int J Equity Health. 2024;23(1):247. doi: 10.1186/s12939-024-02333-x 39580443 PMC11585951

[18] Su W, Du L, Fan Y, Wang P. Equity and efficiency of public hospitals’ health resource allocation in Guangdong Province, China. Int J Equity Health. 2022;21(1):138. doi: 10.1186/s12939-022-01741-1 36138478 PMC9493174

[19] Dai X, Jiang Y, Li Y, Wang X, Wang R, Zhang Y. Evaluation of community basic public health service effect in a city in Inner Mongolia Autonomous Region--based on entropy weight TOPSIS method and RSR fuzzy set. Arch Public Health. 2023;81(1):149. doi: 10.1186/s13690-023-01151-x 37592329 PMC10433667

[20] Zhong X, Wang D-L, Mo L-F, Zhang W, Xiao L-H, Wu X-L, et al. Evaluation of the quality of COVID-19 prevention and control by a novel comprehensive evaluation model in a tertiary general hospital: a prospective observational study. BMC Public Health. 2021;21(1):2022. doi: 10.1186/s12889-021-12032-9 34742268 PMC8571898

[21] Wen Y, Li Y, Zhang Y, Liu B. Comprehensive evaluation of global health cities development levels. Front Public Health. 2024;12:1437647. doi: 10.3389/fpubh.2024.1437647 39091532 PMC11291463

[22] Pan P-J, Hsu N-W, Lee M-J, Lin Y-Y, Tsai C-C, Lin W-S. Physical fitness and its correlation with handgrip strength in active community-dwelling older adults. Sci Rep. 2022;12(1):17227. doi: 10.1038/s41598-022-21736-w 36241763 PMC9568649

[23] Wang L-Y, Hu Z-Y, Chen H-X, Zhou C-F, Tang M-L, Hu X-Y. Differences in regional distribution and inequality in health workforce allocation in hospitals and primary health centers in China: a longitudinal study. Int J Nurs Stud. 2024;157:104816. doi: 10.1016/j.ijnurstu.2024.104816 38824719

[24] Qin C, Liu M, Guo X, Liu J. Human resources in primary health-care institutions before and after the new health-care reform in china from 2003 to 2019: an interrupted time series analysis. Int J Environ Res Public Health. 2022;19(10):6042. doi: 10.3390/ijerph19106042 35627589 PMC9140912

[25] Song C, Fang L, Xie M, Tang Z, Zhang Y, Tian F, et al. Revealing spatiotemporal inequalities, hotspots, and determinants in healthcare resource distribution: insights from hospital beds panel data in 2308 Chinese counties. BMC Public Health. 2024;24(1). doi: 10.1186/s12889-024-17950-y PMC11218403 38336709

[26] Ye J, Feng J, Li X, Qu G, Lei Z, Jiang H, et al. Public trust in general practitioners and its association with primary care contracts: a cross-sectional study of community residents in China. Public Health. 2024;231:55–63. doi: 10.1016/j.puhe.2024.03.014 38626672

[27] Zhao X, Zhang Y, Yang Y, Pan J. Diabetes-related avoidable hospitalisations and its relationship with primary healthcare resourcing in China: a cross-sectional study from Sichuan Province. Health Soc Care Community. 2022;30(4):e1143–56. doi: 10.1111/hsc.13522 34309097

[28] Tan X. A review of China’s national policies to strengthen primary care 2003-2018. Glob Public Health. 2023;18(1):2252049. doi: 10.1080/17441692.2023.2252049 37647350

[29] van Gool K, Mu C, Hall J. Does more investment in primary care improve health system performance?. Health Policy. 2021;125(6):717–24. doi: 10.1016/j.healthpol.2021.03.004 33906796

[30] Li M, Zhang X, Tang H, Zheng H, Long R, Cheng X, et al. Quality of primary health care for chronic diseases in low-resource settings: evidence from a comprehensive study in rural China. PLoS One. 2024;19(7):e0304294. doi: 10.1371/journal.pone.0304294 39052549 PMC11271947

[31] Li M, Tang H, Zheng H, Tian Y, Cheng X, Cheng H, et al. Supporting and retaining competent primary care workforce in low-resource settings: lessons learned from a prospective cohort study. Fam Med Com Health. 2023;11(4):e002421. doi: 10.1136/fmch-2023-002421 PMC10632899 37931977

[32] Yang G, Zhang X, Xu Z, Zhang L. Social medical insurances, choices of medical institutions and the “siphon effect” in the health service market: evidence from 2021 Yangtze river delta region of China. Risk Manag Healthc Policy. 2024;17:1287–99. doi: 10.2147/RMHP.S458178 38770148 PMC11104391

[33] Qian J, Ramesh M. Strengthening primary health care in China: governance and policy challenges. Health Econ Policy Law. 2024;19(1):57–72. doi: 10.1017/S1744133123000257 37846025

[34] Huang S, Yin A, Liu Q, Sun X. Can the implementation of family doctor contracted service enable the elderly to utilize primary health care services more equally? empirical evidence from Shandong, China. BMC Prim Care. 2022;23(1):31. doi: 10.1186/s12875-022-01630-0 35189808 PMC8862204

[35] Wang W, Zhang J, Loban K, Wei X. High performing primary health care organizations from patient perspective: a qualitative study in China. Glob Health Res Policy. 2023;8(1):31. doi: 10.1186/s41256-023-00315-0 37544999 PMC10405398

[36] Fu P, Wang Y, Zhao D, Yang S, Zhou C. Does contracting family doctor promote primary healthcare utilization among older adults? - evidence from a difference-in-differences analysis. BMC Geriatr. 2024;24(1). doi: 10.1186/s12877-024-05336-z PMC11385809 39256643

[37] Xu X, Huang J, Zhao X, Luo Y, Wang L, Ge Y, et al. Trends in the mobility of primary healthcare human resources in underdeveloped regions of western China from 2000 to 2021: evidence from nanning. BMC Prim Care. 2024;25(1). doi: 10.1186/s12875-024-02403-7 PMC11071274 38711072

[38] Cai C, Hone T, Millett C. The heterogeneous effects of China’s hierarchical medical system reforms on health service utilisation and health outcomes among elderly populations: a longitudinal quasi-experimental study. Lancet. 2023;402 Suppl 1:S30. doi: 10.1016/S0140-6736(23)02141-4 37997071

[39] Zhang W, Su M, Li D, Yang F, Li Z. The association between family doctor contract services and the health of middle-aged and older people in China: an instrumental variables analysis. Sci Rep. 2024;14(1):16229. doi: 10.1038/s41598-024-65621-0 39004624 PMC11247085

[40] Liu T, Li J, Chen J, Yang S. Regional differences and influencing factors of allocation efficiency of rural public health resources in China. Healthcare (Basel). 2020;8(3):270. doi: 10.3390/healthcare8030270 32823864 PMC7551190

[41] Jia P, Wang Y, Yang M, Wang L, Yang X, Shi X, et al. Inequalities of spatial primary healthcare accessibility in China. Soc Sci Med. 2022;314:115458. doi: 10.1016/j.socscimed.2022.115458 36279792

[42] Li L, Zhu L, Zhou X, Zeng G, Huang H, Gan Y, et al. Patients’ trust and associated factors among primary care institutions in China: a cross-sectional study. BMC Prim Care. 2022;23(1):109. doi: 10.1186/s12875-022-01709-8 35524197 PMC9075926

[43] Fu L, Han J, Xu K, Pei T, Zhang R. Incentivizing primary care utilization in China: the impact of health insurance coverage on health-seeking behaviour. Health Promot Int. 2024;39(5):daae115. doi: 10.1093/heapro/daae115 39243132

[44] Dong E, Xu J, Sun X, Xu T, Zhang L, Wang T. Differences in regional distribution and inequality in health-resource allocation on institutions, beds, and workforce: a longitudinal study in China. Arch Public Health. 2021;79(1):78. doi: 10.1186/s13690-021-00597-1 34001268 PMC8130126

